# The association of tadalafil exposure with lower rates of major adverse cardiovascular events and mortality in a general population of men with erectile dysfunction

**DOI:** 10.1002/clc.24234

**Published:** 2024-02-20

**Authors:** Robert A. Kloner, Eric Stanek, Karishma Desai, Christopher L. Crowe, Kathryn Paige Ball, Aaron Haynes, Raymond C. Rosen

**Affiliations:** ^1^ Huntington Medical Research Institutes Pasadena California USA; ^2^ Keck School of Medicine of University of Southern California Los Angeles California USA; ^3^ Elevance Health Inc. Indianapolis Indiana USA; ^4^ Carelon Research Inc. Wilmington Delaware USA; ^5^ Department of Psychiatry and Behavioral Sciences University of California, San Francisco San Francisco California USA

**Keywords:** erectile dysfunction, major adverse cardiovascular events, tadalafil, total mortality

## Abstract

**Background:**

Tadalafil is a long‐acting phosphodiesterase‐5 inhibitor (PDE‐5i) indicated for erectile dysfunction (ED).

**Hypothesis:**

Our hypothesis was that tadalafil will reduce the risk of major adverse cardiovascular events (MACE: composite of cardiovascular death, myocardial infarction, coronary revascularization, unstable angina, heart failure, stroke) and all‐cause death in men with ED.

**Methods:**

A retrospective observational cohort study was conducted in a large US commercial insurance claims database in men with a diagnosis of ED without prior MACE within 1 year. The exposed group (*n* = 8156) had ≥1 claim for tadalafil; the unexposed group (*n* = 21 012) had no claims for any PDE‐5i.

**Results:**

Primary outcome was MACE; secondary outcome was all‐cause death. Groups were matched for cardiovascular risk factors, including preventive therapy. Over a mean follow‐up of 37 months for the exposed group and 29 months for the unexposed group, adjusted rates of MACE were 19% lower in men exposed to tadalafil versus those unexposed to any PDE‐5i (hazard ratio [HR] = 0.81; 95% confidence intervals [CI] = 0.70−0.94; *p* = .007). Tadalafil exposure was associated with lower adjusted rates of coronary revascularization (HR = 0.69; 95% CI = 0.52−0.90; *p* = .006); unstable angina (HR = 0.55; 95% CI = 0.37−0.81; *p* = .003); and cardiovascular‐related mortality (HR = 0.45; CI = 0.22−0.93; *p* = .032). Overall mortality rate was 44% lower in men exposed to tadalafil (HR = 0.56; CI = 0.43−0.74; *p* < .001). Men in the highest quartile of tadalafil exposure had the lowest rates of MACE (HR: 0.40; 95% CI: 0.28−0.58; *p* < .001) compared to lowest exposure quartile.

**Conclusion:**

In men with ED, exposure to tadalafil was associated with significant and clinically meaningful lower rates of MACE and overall mortality.

## Graphical abstract

Forest plot demonstrating that men with erectile dysfunction (ED) who were exposed to tadalafil (*N* = 8156), had significantly lower adjusted rates of major adverse cardiovascular events (hazard ratio [HR]: 0.81), coronary revascularization (HR: 0.69), unstable angina (HR: 0.55), cardiovascular related deaths (HR: 0.45), and all cause death (HR: 0.56), compared to closely matched men with ED not exposed to tadalafil or other PDE‐5 inhibitors (*N* = 21 012). Py, patient‐years.

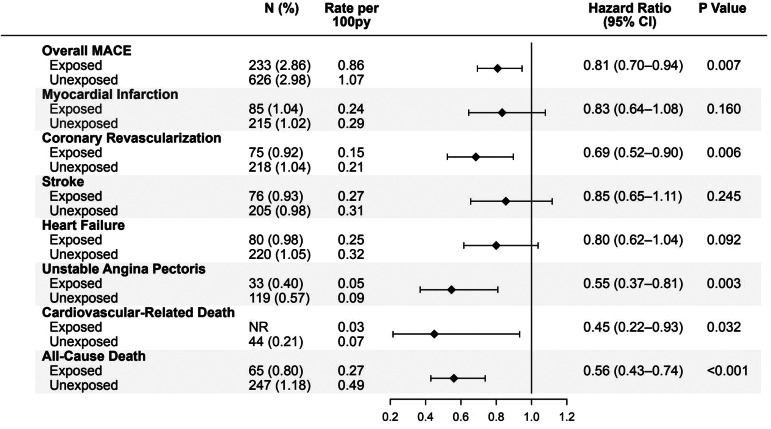

**Abbreviations**
CI: confidence interval
ED: erectile dysfunction
HIRD: Healthcare Integrated Research Database
HR: hazard ratio
IRB: Institutional Review Board
MACE: major adverse cardiovascular events
NDI: national death index
PDE‐5i: phosphodiesterase‐5 inhibitors

## 1 INTRODUCTION

The phosphodiesterase‐5 inhibitors (PDE‐5is) have been shown to be effective for treatment of erectile dysfunction (ED). Recent retrospective studies have suggested that PDE‐5i use may be associated with reduced major adverse cardiovascular events (MACE) and mortality, especially in subgroups of men with diabetes; some have suggested a dose effect.^1^, ^2^, ^3^, ^4^, ^5^, ^6^, ^7^ Most studies have not focused on a single PDE‐5i in a general population of men with ED. If exposure is an important feature, then we would predict that a long acting PDE‐5i, such as tadalafil (17.5‐hour half‐life) may have a pronounced association between exposure and lower rates of MACE. Therefore, the purpose of this study was to assess the relationship between tadalafil use and the extent of exposure with cardiovascular outcomes (MACE: composite of cardiovascular death, or hospitalization for myocardial infarction, coronary revascularization, unstable angina, heart failure, and stroke) and mortality from any cause in men with ED.

## 2 METHODS

### 2.1 Study design

This is a retrospective observational cohort study using a large commercial administrative medical and pharmacy claims database, Healthcare Integrated Research Database (HIRD®). The HIRD represents claims information from the largest commercially insured population in the United States and includes the lines of business such as health maintenance organizations, point of service plans, preferred provider organizations, Medicare advantage, and consumer directed health plans and indemnity plans. The data were linked to national death index (NDI), the federal database for all recorded deaths in the United States, maintained by the Centers for Disease Control to capture death and cause of death data.^8^ The study population was composed of men with both medical and pharmacy coverage in the database during the study period.

We identified men aged 18 years or older, with ≥1 diagnosis of ED between January 1, 2006 and October 31, 2020 (study period). The exposed group consisted of men who newly used the PDE‐5i, tadalafil, after a diagnosis of ED during the patient identification period (January 1, 2007 to October 31, 2020), without any PDE‐5i claim or MACE in the baseline period (12 months before the index date). The first claim date of tadalafil was defined as the index date of the exposed group. The unexposed group (controls) were patients with a diagnosis of ED, but without tadalafil or other PDE‐5i use in the study period. Index date of the unexposed group was the date of first pharmacy claim of any treatment closest to a randomly selected date based on the distribution of days between first ED diagnosis and the index date among exposed. The baseline period was used to capture any baseline covariates that later were used to adjust for any baseline differences. The follow‐up period started at the index date until patients were censored, defined as end of continuous enrollment, death, claim of outcomes (MACE), study end date, whichever occurred earlier.

The primary outcome was MACE, defined above. Secondary outcomes were all‐cause mortality and the individual components of MACE. Objective 1 was to determine the association of tadalafil exposure with MACE and overall mortality among the entire cohort of men with ED. Objective 2 was to evaluate the association of tadalafil with MACE and overall mortality among men with ED and no overt coronary artery disease, but with the diagnosis of significant cardiovascular risk factors as previously described.^1^ Objective 3 was to establish the relationship between varying levels of exposure to tadalafil, measured by the number of tablets dispensed during follow‐up, and MACE and mortality in the entire cohort of exposed men with ED. Objective 4 repeated objective 1 among the subgroups of men with ED and type 2 diabetes or men with baseline overt coronary artery disease. Data on the overall cohort of men on any PDE‐5i has been previously presented,^1^ but data on the specific long‐acting agent tadalafil has not previously been analyzed.

### 2.2 Statistics

Exact matching (matched up to 1:4 on baseline risk variables) plus multivariable adjustments were selected given its ability to account for residual imbalances that remain after matching alone in claims‐based analyses. For multivariable statistics, Cox proportional hazards models were used to report associations between tadalafil use and outcomes.^1^ Hazard ratios (HR) along with 95% confidence intervals (CI) were computed to document the association between exposure and outcomes. In addition, Kaplan−Meier curves were plotted to visualize the association and survival for variable length of follow‐up period. The protocol received a waiver of authorization from the Huntington Medical Research Institute's Institutional Review Board (IRB). A Health Insurance Portability and Accountability Act Waiver of Authorization was obtained from Carelon Research's IRB for conducting linkage to the NDI.

## 3 RESULTS

Baseline demographics are shown in Table 1. There were 8156 men in the exposed group and 21 012 in the nonexposed group. Mean age was 51.9 ± 10.2 years (mean ± SD) in the exposed group and 51.5 ± 10.9 in the unexposed group. Participants were matched for baseline common risk factors and medicines including: smoking indicators, ischemic heart disease, diabetes, hypertension, hypercholesterolemia, antiplatelet agents, antihypertensives, and statins.

**Table 1:** Baseline clinical conditions and treatment use in patients with erectile dysfunction.

	Exposed group	Unexposed group	*p* Value^a^
Number of patients, *n* (%)	8156 (28.0)	21 012 (72.0)	—
Current smoking,^b^ *n* (%)Matched			
Yes	442 (5.4)	1132 (5.4)	—
No	7714 (94.6)	19 880 (94.6)	
Clinical comorbidities, *n* (%)			
Ischemic heart disease/coronary artery diseaseMatched	144 (1.8)	312 (1.5)	—
Type 2 diabetes mellitusMatched	675 (8.3)	1644 (7.8)	—
HypertensionMatched	2745 (33.7)	6845 (32.6)	—
Hypercholesterolemia, dyslipidemiaMatched	2998 (36.8)	7366 (35.1)	—
Atrial fibrillation	160 (2.0)	312 (1.5)	**.002**
Ventricular arrhythmia	27 (0.3)	53 (0.3)	.233
Peripheral arterial disease	96 (1.2)	229 (1.1)	.962
Benign prostatic hypertrophy	1740 (21.3)	3331 (15.9)	**<.001**
Chronic kidney disease	126 (1.5)	324 (1.5)	.936
Hypogonadism	932 (11.4)	2260 (10.8)	.331
Postprocedural testicular hypofunction	≤10	11 (0.1)	.056
Hypogonadotropic hypogonadism/hypopituitarism	≤10	44 (0.2)	**.044**
Treatment use in baseline period, *n* (%)			
Implantable cardioverter‐defibrillator	≤10	≤10	NA
Cardiac resynchronization pacemakers	≤10	≤10	NA
Warfarin, direct oral anticoagulants	129 (1.6)	316 (1.5)	.776
Digoxin	18 (0.2)	34 (0.2)	.305
Antiplatelets including acetylsalicylic acidMatched	19 (0.2)	40 (0.2)	—
Antianginals			
Ranolazine	≤10	≤10	.257
Nitrates—short‐ and long‐acting	40 (0.5)	99 (0.5)	.655
StatinsMatched			
High‐intensity	383 (4.7)	962 (4.6)	.193
Moderate‐low intensity	1521 (18.6)	3719 (17.7)	**.023**
Non‐statin lipid lowering agents			
PCSK9 inhibitors	≤10	≤10	.480
Ezetimibe/cholesterol absorption inhibitors	133 (1.6)	250 (1.2)	**.029**
Fibrates	258 (3.2)	608 (2.9)	.144
Niacin	64 (0.8)	132 (0.6)	.670
EPA/DHA	65 (0.8)	132 (0.6)	.111
Adenosine triphosphate‐citrate lyase inhibitors	≤10	≤10	NA
Bile acid sequestrants	41 (0.5)	85 (0.4)	.411
Anti‐hypertensivesMatched			
Angiotensin‐converting enzyme inhibitors	1368 (16.8)	3497 (16.6)	.792
Angiotensin receptor blockers	815 (10.0)	1786 (8.5)	**<.001**
Angiotensin receptor‐neprilysin inhibitor	≤10	≤10	NA
Beta‐blockers	697 (8.5)	1874 (8.9)	**.004**
Calcium channel blockers	781 (9.6)	2014 (9.6)	.118
Renin inhibitor	≤10	16 (0.1)	.411
Aldosterone receptor modulators	82 (1.0)	261 (1.2)	**.005**
Antiadrenergics	151 (1.9)	392 (1.9)	.087
Vasodilators	15 (0.2)	55 (0.3)	.225
Diuretics	542 (6.6)	1429 (6.8)	.149
Type 2 diabetes mellitus therapy			
Metformin	435 (5.3)	1051 (5.0)	**.045**
Dipeptidyl peptidase 4 inhibitors	109 (1.3)	237 (1.1)	**.033**
Glucagon‐like peptide‐1 receptor agonists	63 (0.8)	134 (0.6)	**.035**
Sodium‐glucose cotransporter‐2 inhibitors	41 (0.5)	96 (0.5)	.347
Sulphonylureas	206 (2.5)	550 (2.6)	.236
Thiazolidinediones	105 (1.3)	202 (1.0)	**.008**
Insulin	158 (1.9)	398 (1.9)	.355
Other^c^	13 (0.2)	22 (0.1)	.109
Androgen/testosterone replacement therapy	525 (6.4)	1320 (6.3)	.090
Non‐PDE5i ED therapy (Rx and nonpharmacologic)			
Alprostadil (injectable and MUSE)	21 (0.3)	22 (0.1)	**.001**
Other injectables (papaverine, phentolamine)	≤10	15 (0.1)	.853
Nonpharmacologic (implant/pump, vacuum, revascularization)	45 (0.6)	73 (0.3)	**.008**

*Note*: Bold values indicate statistically significant values.

Abbreviations: ED, erectile dysfunction; EPA/DHA, eicosapentaenoic acid/docosahexaenoic acid; MUSE, medicated urethral system for erections; n, number; PDE5i; phosphodiesterase‐5 inhibitors; %, percentage.

^a^ *p* Values calculated using McNemar's tests.

^b^ Based on Desai et al. published algorithm.

^c^ Amylin analogs, meglitinide analogs, alpha‐glucosidase inhibitors, dopamine receptor agonists.

**^Matched^ :**the variable was used to match the exposed and unexposed group for the multivariate analyses. Patients matched based on days between ED diagnosis and index date, age (±3 years), smokers, any use of statin or anti‐hypertensive or antiplatelet medication, and presence of ischemic heart disease or coronary artery disease, hypercholesterolemia or dyslipidemia, hypertension, and Type 2 diabetes mellitus.

Baseline demographics in exposed versus unexposed groups. Supra‐script for matched values.

**Figure 1:**
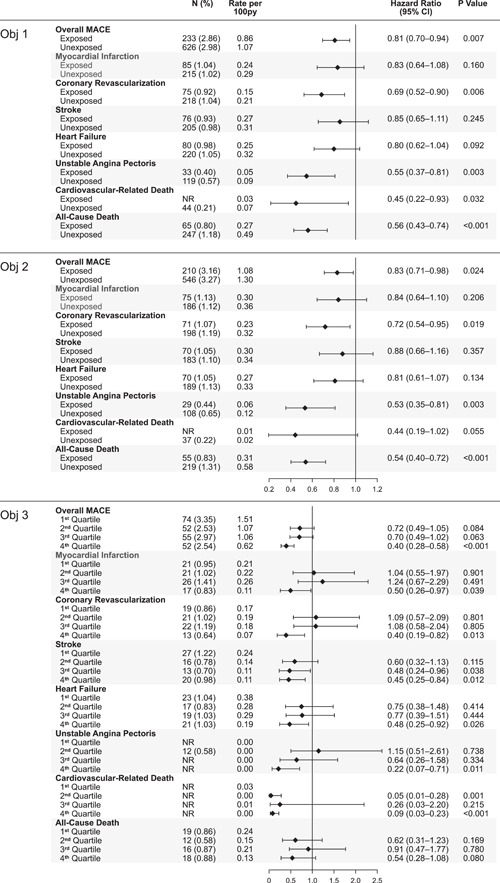
Forest plots for outcomes including rate per 100py (patient‐years), hazard ratios, confidence intervals, *p* values. Top panel: Forest plots showing overall MACE, components of MACE, and all cause death for the general cohort of men with ED. Objective (Obj) 1, exposed and unexposed to tadalafil. Middle panel: Forest plots for outcomes of men without known coronary artery disease but with risk factors for coronary artery disease (Obj 2). Bottom panel: Forest plots showing outcomes by dose. The lowest dose is the first quartile and the highest dose the fourth quartile (Obj 3). The first quartile represents 6.0 ± 1.3 tablets prescribed during the study period; second quartile represents 16.1 ± 4.9 tablets; third quartile represents 42.2 ± 12.2 tablets; and fourth quartile represents 288.6 ± 327.6 tablets. ED, erectile dysfunction; MACE, major adverse cardiovascular events. NR, not reportable, total count less than or equal to 10.

**Figure 2:**
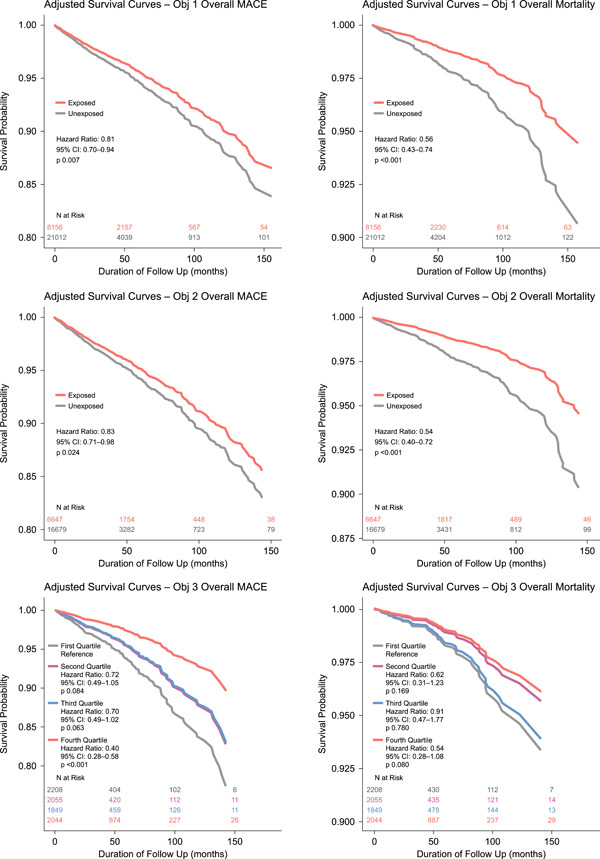
Adjusted Kaplan−Meier survival curves showing survival free of MACE or mortality due to any cause. Top left: Overall MACE for objective 1; top right: Overall mortality for objective 1; middle panel left: MACE for objective 2; middle panel right: Overall mortality for objective 2; bottom panel left: MACE for objective 3 showing outcomes comparing Quartiles 2, 3, and 4 to 1; bottom panel right: Overall mortality showing outcomes comparing quartiles. MACE, major adverse cardiovascular events.

Key outcomes with HRs, CIs, *p* values, and Kaplan−Meier curves are shown in Figures 1 and 2. Objective 1: Over a mean follow‐up of 37 months for the exposed group and 29 months for the unexposed group, adjusted rate of MACE was 19% lower in the general cohort of men with ED exposed to tadalafil versus those unexposed to PDE‐5 inhibitors (HR = 0.81; 95% CI = 0.70−0.94; *p* = .007). Tadalafil exposure was associated with lower adjusted rates of coronary revascularization (HR = 0.69; 95% CI = 0.52−0.90; *p* = .006); unstable angina (HR = 0.55; 95% CI = 0.37−0.81; *p* = .003); and cardiovascular‐related mortality (HR = 0.45; CI = 0.22−0.93; *p* = .032). Overall mortality was 44% lower in men exposed to tadalafil (HR = 0.56; CI = 0.43−0.74; *p* < .001) versus unexposed. Objective 2: Men with ED who did not have known coronary artery disease but had risk factors also showed similar results. Objective 3: Men in the general cohort, in the highest quartile of tadalafil exposure had the lowest incidence of MACE (HR: 0.40; 95% CI: 0.28−0.58; *p* < .001) versus the lowest exposure quartile. Higher tadalafil exposure was also associated with lower rates of myocardial infarction and stroke as well as other components of MACE. Objective 4: Tadalafil exposure was not associated with significantly lower rates of MACE or mortality in the subgroups of men with Type 2 diabetes (MACE: HR = 0.77, 95% CI = 0.53−1.12, *p* = .173; mortality: HR = 0.66, 95% CI = 0.33–1.31, *p* = .233) or known coronary artery disease (MACE: HR = 0.66, 95% CI = 0.32−1.34, *p* = .247; mortality: HR = 1.40, 95% CI = 0.57–3.42, *p* = .467).

## 4 DISCUSSION

In a large population of US men with ED, exposure to tadalafil was associated with significant and clinically meaningful lower rates of MACE, cardiovascular death, unstable angina, need for revascularization, and overall mortality. Highest doses were associated with better outcomes. To the best of our knowledge this is the first study to look at the association between exposure to the specific long‐acting PDE‐5 inhibitor, tadalafil, and cardiovascular outcomes in a large population of men with ED in the United States. These data add to a growing number of observational studies supporting a cardioprotective effect of PDE‐5 inhibitors.^1^, ^2^, ^3^, ^4^, ^5^, ^6^, ^7^ In our study, the benefit was most pronounced in a general cohort of men with ED rather than in subsets with diabetes or coronary artery disease^2^, ^3^, ^4^, ^5^; most likely related to smaller sample sizes in these subsets from our study. Of note the adjusted HR for these two subgroups still favored tadalafil (HR of 0.77 for MACE in the diabetic subgroup and 0.66 in the coronary artery subgroup comparing exposure to nonexposure) and were of similar magnitude as those observed in the overall cohort analysis in Objective 1; but these HR did not reach statistical significance with wider CIs, likely due to limited statistical power stemming from low event rates and smaller sample sizes. For example, while the overall cohort sizes of men with ED included 8156 patients in the exposed group and 21 012 in the unexposed group; in the diabetic subgroup there were only 675 exposed patients and 1644 unexposed patients; in the coronary artery disease subgroup, there were only 144 exposed patients and 312 unexposed patients.

Strengths of this study include the large database, the length of follow‐up, the adjustment for confounding risk factors and medicines. Weaknesses include the retrospective nature of the study, the possibility of unknown confounding variables and the unknown issue of whether the drug conferred direct benefit or whether sexual activity promoted by the drug led to a benefit. However, in one previous study, while PDE5 inhibitors were associated with a reduction in cardiovascular events, another drug that is used to treat ED (alprostadil) did not have a benefit on cardiac outcomes, suggesting that the PDE5 inhibitors themselves had a cardioprotective effect.^4^ However, alprostadil is a local injectable drug, typically used intermittently and likely in men with more severe ED; so, the comparison between alprostadil and more chronic use of PDE‐5 inhibitors should be considered as exploratory. Another weakness of our study is that we did not compare tadalafil directly with short acting agents in the class of drugs of PDE‐5 inhibitors. Such a comparison would be worth pursuing in future studies, but was beyond the scope of our current study and central hypothesis, which was limited to tadalafil exposure versus no tadalafil exposure.

A crucial next step is the design of prospective, randomized, controlled studies to further explore this potential benefit in men and women and clinical applications. Our study was not designed to determine the mechanism of action of the benefit of tadalafil. However, several theories have been proposed to explain the potential cardioprotective effects of the PDE‐5 inhibitors. These include an improvement in endothelial function, vasodilation of systemic blood vessels resulting in small reductions in blood pressure and afterload, a direct protective effect on myocardial cells, an anti‐inflammatory property, and an antiplatelet effect.^1^ Future studies are needed to further explore the mechanism/s whereby PDE‐5 inhibitors may have cardioprotective effects.

## 5 CONCLUSIONS

The use of tadalafil in men with ED was associated with lower rates of MACE as well as total mortality and there appears to be a dose response. Prospective randomized placebo‐controlled trials in both men and women are needed in the future.

## CONFLICT OF INTEREST STATEMENT

Sanofi was not involved with data collection or data analysis or in drafting of the manuscript. Dr. Robert A. Kloner is a paid consultant to Sanofi but did not receive consulting fees for this project. Dr. Raymond Rosen received a consulting fee from HMRI. The other coauthors were employees of Carelon which received a subaward from HMRI for the current study.

## Data Availability

The data that support the findings of this study are available from the corresponding author upon reasonable request.

## References

[1] Kloner RA , Stanek E , Crowe CL , et al. Effect of phosphodiesterase type 5 inhibitors on major adverse cardiovascular events and overall mortality in a large nationwide cohort of men with erectile dysfunction and cardiovascular risk factors: a retrospective, observational study based on healthcare claims and national death index data. J Sex Med. 2023;20:38‐48. 36897243 10.1093/jsxmed/qdac005

[2] Anderson SG , Hutchings DC , Woodward M , et al. Phosphodiesterase type‐5 inhibitor use in type 2 diabetes is associated with a reduction in all‐cause mortality. Heart. 2016;102:1750‐1756. 27465053 10.1136/heartjnl-2015-309223 PMC5099221

[3] Andersson DP , Trolle Lagerros Y , Grotta A , Bellocco R , Lehtihet M , Holzmann MJ . Association between treatment for erectile dysfunction and death or cardiovascular outcomes after myocardial infarction. Heart. 2017;103:1264‐1270. 28280146 10.1136/heartjnl-2016-310746 PMC5537549

[4] Andersson DP , Landucci L , Lagerros YT , et al. Association of phosphodiesterase−5 inhibitors versus alprostadil with survival in men with coronary artery disease. J Am Coll Cardiol. 2021;77:1535‐1550. 33766260 10.1016/j.jacc.2021.01.045

[5] Hackett G , Jones PW , Strange RC , Ramachandran S . Statin, testosterone and phosphodiesterase 5‐inhibitor treatments and age related mortality in diabetes. World J Diabetes. 2017;8:104‐111. 28344753 10.4239/wjd.v8.i3.104 PMC5348622

[6] Wilton KM , Achenbach SJ , Davis 3rd, JM , Myasoedova E , Matteson EL , Crowson CS . Erectile dysfunction and cardiovascular risk in men with rheumatoid arthritis: a population‐based cohort study. J Rheumatol. 2021;48:1641‐1647. 33452166 10.3899/jrheum.201226 PMC8280238

[7] Vestergaard N , Søgaard P , Torp‐Pedersen C , Aasbjerg K . Relationship between treatment of erectile dysfunction and future risk of cardiovascular disease: a nationwide cohort study. Eur J Preventive Cardiol. 2017;24:1498‐1505. 10.1177/2047487317718082 28656785

[8] MacMahon B . The national death index. Am J Public Health. 1983;73:1247‐1248. 10.2105/ajph.73.11.1247 6625026 PMC1651137

